# Connexin43 in Musculoskeletal System: New Targets for Development and Disease Progression

**DOI:** 10.14336/AD.2022.0421

**Published:** 2022-12-01

**Authors:** Senbo An, Shengyuan Zheng, Zijun Cai, Siyu Chen, Chen Wang, Yusheng Li, Zhenhan Deng

**Affiliations:** ^1^Department of Orthopaedics, Shandong Provincial Hospital Affiliated to Shandong First Medical University, Jinan, Shandong, China.; ^1^Department of Orthopedics, Xiangya Hospital, Central South University, Changsha, Hunan, China.; ^1^Department of Sports Medicine, The First Affiliated Hospital of Shenzhen University, Shenzhen Second People's Hospital, Shenzhen, Guangdong, China.; ^1^National Clinical Research Center for Geriatric Disorders, Xiangya Hospital, Central South University, Changsha, Hunan, China.; ^1^Department of Clinical Medicine, Xiangya Medicine School, Central South University, Changsha, Hunan, China.

**Keywords:** liver, Connexin43, gap junction, hemichannel, musculoskeletal system, rheumatoid arthritis, osteoporosis, osteoarthritis

## Abstract

Connexin43, which is the most highly expressed connexin subtype in the musculoskeletal system, exists in a variety of bone cells, synovial tissue, and cartilage tissue. Connexin43 has been suggested to be a key regulator of bone homeostasis. Studies have shown aberrant Connexin43 expression in musculoskeletal disorders, such as osteoporosis, osteoarthritis, and rheumatoid arthritis. During cellular activities, Connexin43 can participate in the formation of functionally specific gap junctions and hemichannels and can exert independent cellular regulatory and signaling functions through special C-termini. The critical role of Connexin43 in physiological development and disease progression has been gradually revealed. In this article, the function of Connexin43 in musculoskeletal tissues is summarized, revealing the potential role of Connexin43 as a key target in the treatment of related bone and muscle disorders and the need for further discovery.

## 1. Introduction

Connexin43 (Cx43) is a member of the connexin family, which includes nearly 21 genes identified in the human body, each of which is named according to its molecular weight. For example, the connexin with a molecular weight of 43 kD is named Cx43. Cx43 is very similar in structure to other connexins but differs in biological functional properties [1]. The dissimilarities are mainly manifested in the permeability of channels composed of Connexins for various ions and signaling messengers, such as Ca^2+^, cAMP and cGMP [2,3]. Six identical or different connexins can constitute connexons. The functional oligomer is usually located on the plasma membrane and exists in pairs to form gap junctions (GJs) between adjacent cells or in single cells to form hemichannels (HCs) as material exchange channels. Both GJs and HCs are vital gated channels and are permeable to specific substances depending on selectivity. Additionally, based on different stimuli, the channels can be divided into voltage gated and chemical gated channels [4]. The normal life activities of many cell types are linked to GJs and HCs and depend on them to regulate key activities, such as cell growth, migration and gene expression [5].

Connexons are distributed in numerous organs and tissues and are critical in certain physiological activities, such as organogenesis and homeostasis. Cx43 has been gradually highlighted in recent research as an important regulator in the musculoskeletal system based on the discovery of its hemichannel and extracellular mechano-sensing mechanism, especially in bone development and joint diseases [6], such as rheumatoid arthritis (RA), osteoarthritis (OA), osteoporosis and occulodentodigital dysplasia (ODDD) [7-10]. Therefore, Cx43 has the potential to be a marker for bone disease diagnosis or a novel target for future treatment strategies.

In this article, we outline the basic structure and function of Cx43, including components of GJs and HCs in major joint tissues, such as synovial tissue, cartilage tissue and bone tissue. We pay special attention to the newly discovered role of Cx43 in the perception of bone mechanical loading. In addition, we focus on the impact of Cx43 dysregulation in musculoskeletal diseases and discuss the potential of Cx43 as a novel target for therapeutic strategies.

## 2. Basic molecular structure and function of Cx43

**Figure 1.**
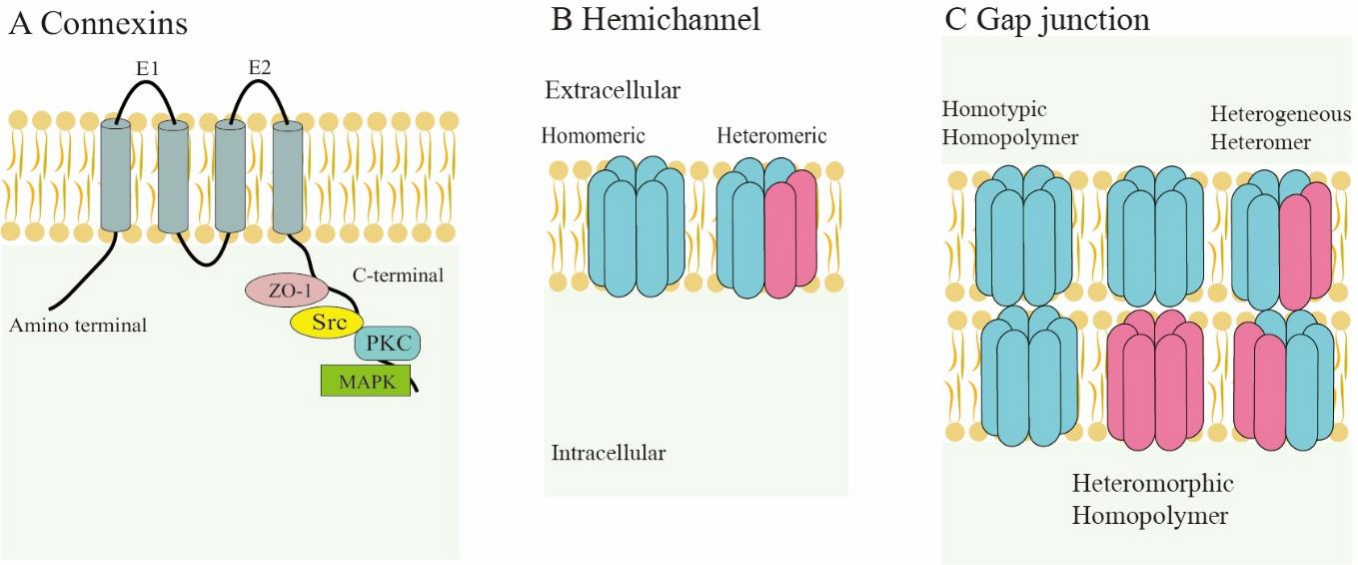
Structure of connexin43, hemichannels and gap junctions. (A) Cx43 is composed of 4 segment structures, its cytoplasmic side has an amino terminus, a special function carboxy terminus and a cytoplasmic loop domain, and there are two loop domains on the extracellular side (E1 and E2 respectively). (B) Hemichannels can be divided into two types, homomeric and heteromeric. (C) Gap junctions can be divided into homotypic homopolymer, heteromorphic homopolymer, heterogeneous heteromer.

Cx43, one of the most important connexins, can form gap junctions and hemichannels and functions as a critical mediator in intercellular communication. Cx43 is composed of multiple segment structures; its cytoplasmic side has an amino terminus, a special function carboxy terminus and a cytoplasmic loop domain, and two loop domains are present on the extracellular side (E1 and E2). Four transmembrane segments (TM1, TM2, TM3 and TM4) connect the two sides of the plasma membrane portion of the intermediate structure [11]. Cx43 has been demonstrated to participate in many cell behaviors [12], however, aside from the C-terminus (CT), the other portions of Cx43 have not been found to be related to core processes in cell behaviors. The CT of Cx43 mainly functions as a scaffold that interacts with protein kinase C (PKC), mitogen-activated protein kinase (MAPK), β-catenin, integrins, the proto-oncogene tyrosine-protein kinase Src and the tight junction protein ZO-1 (Fig. 1A) [6,13-16].

Cx43 is normally located at the plasma membrane and sometimes is present in the Golgi, endoplasmic reticulum and nucleus [12]. Plasmalemma Cx43 mainly forms hexameric connexons. The structure of connexons can be classified as homomeric or heteromeric based on the type of polymerization, similar to hemichannels (Fig. 1B) and GJ channels [11] (Fig. 1C). Compared with GJs sandwiched between adjoining plasma membranes, hemichannels allow the exchange of substances between the cellular matrix and extracellular environment [4]. The mode of operation for these two channel types is similar to that of a normal channel on the membrane, and the gate control responds to specific stimuli. GJs are permeable to molecules < 1 kD in size, including Na^+^, K^+^, lactate, glucose-6-phosphate, signaling molecules and ATP; however, with similar permeability, HCs mainly control the release of molecules, such as ATP, PGE2, NAD^+^, glutathione, glutamate, and K^+^, from the cytoplasm [17]. Through its intracellular CT and functional connexons, Cx43 regulates many cellular activities. However, no obvious conclusions have been drawn about the function of intracellular Cx43, and there is speculation that the appearance of intracellular Cx43 is an intermediate process in Cx43 translocation to the cell membrane after expression.

Considerable evidence suggests that Cx43 plays significant roles in numerous organs and tissues, such as the heart, lung, and nervous system [18-21]; moreover, independent of GJ structures, Cx43 is critical in regulating fetal nervous system development during embryogenesis [12,18,22]. All these functions are likely to benefit from its special CT. The CT of Cx43 is controlled via wide-ranging posttranslational modifications, such as phosphorylation of serine, tyrosine and threonine residues [23]; acetylation [24]; and S-nitrosylation [25], which modulate the intracellular transport and functions of hemichannels and the permeability of GJs [26,27]. Several functional domains constitute the CT of Cx43 and can regulate intracellular signal transmission and substance interactions. Genetic knockout of Cx43 strongly affects the cellular transcriptome and results in deterioration of many biological processes, including cell adhesion, proliferation, differentiation and signaling [28].

## 3. Cx43 in physiological musculoskeletal system

### 3.1 Cx43 in development of bone

The connexin family is distributed throughout the musculoskeletal system and regulates the activities of bone, synovium, cartilage, and skeletal muscle by modulating the permeability of connexons [29]. An increasing number of studies have suggested that connexons have a great influence on cells that are crucial to bone development. For example, osteocytes can respond to fluid shear by opening Cx43-HCs to release PGE2 and ultimately promote new bone synthesis [30,31]. Other less distributed Cx members are also important to musculoskeletal activities, such as Cx37 and Cx45 in the osteoblast plasma membrane and Cx46, which was identified as a component of monomeric structures in the Golgi of osteoblasts [32-34]. Studies have shown that the appearance of Cx37 in osteocytes is necessary for the differentiation of osteoclasts [35,36]. Overall, Cx43 is the most significant connexin in the musculoskeletal system, regulating a complicated downstream network and participating in many physiological processes in bone tissue cells, synovial tissue, and articular cartilage [9,10]. Cx43 is critical in skeletal development and growth. Cx43 can expedite skeletal growth by controlling the activity of Runt-related transcription factor 2 (Runx2), a master transcriptional regulator of osteoblastogenesis [37]. A contrast experiment reported by Arum M. Buo et al. showed conspicuous changes in cortical bone and cranial regions and marked increases in cross-sectional area, endosteal and periosteal bone perimeter, and porosity in Gja1 (the Cx43 gene in mice)^+/-^ Runx2^+/-^ mice compared with control mice [38]. ODDD (Gja1 mutation disease) patients usually have characteristic craniofacial abnormalities, aplastic or hypoplastic middle phalanges, syndactyly, and broad tubular long bones [39,40]. Interestingly, the adult skeleton can also be affected by the absence of this connexin according to studies with tissue-specific deletion of Cx43 [41,45-47]. These results suggest that Cx43 is necessary for normal skeletal development and is involved in cellular communication and regulatory activities.

#### 3.1.1 Cx43 in osteoblasts

Cx43 influences bone cell differentiation. According to some studies, the presence of Cx43 is strongly related to osteoblast differentiation, and knockout of Cx43 can reduce differentiation [48-50]. However, the detailed pattern is not truly known from previous reports and remains debatable because Gja1^-/-^ mice showed the opposite consequence, namely, increased periosteal bone formation [45,51]. Cx43 may have a more complex mode of action in regulation, as the Cre-lox recombination results directly targeting the opposite phenomenon showed that the function of Cx43 in osteoblast differentiation is not essential at every stage. For example, early loss of Cx43 can have a greater impact on cell differentiation and extracellular matrix mineralization, while late loss has no significant effect [51,52]. A more detailed report showed that Cx43 affects signal transduction and gene expression in many osteoblasts through its CT. When the Cx43 gene was knocked out, extracellular osteogenesis, intracellular communication, and the expression of specific genes in osteoblasts were significantly reduced, and the entire bone tissue changed dramatically [53].

**Figure 2.**
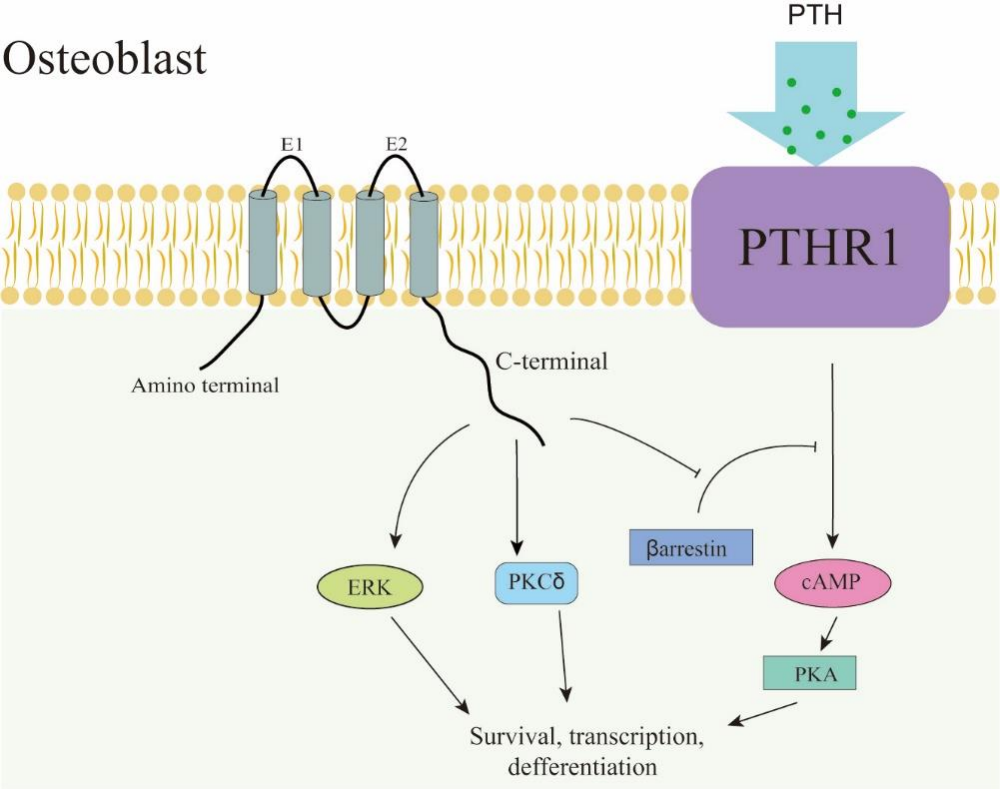
Cx43 can regulate the survival, differentiation, and gene transcription of osteoblasts through ERK and PKCδ signaling pathways in osteoblasts. It can also inhibit βarrestin and have a permissive effect on PTH.

In addition to modulating the transmission of second messengers between cells via GJs, Cx43 can also regulate osteoblastogenesis and cell survival by positively triggering signal transduction cascades. Increasing Cx43 in osteoblasts was found to increase extracellular signal regulated kinase (ERK) signaling [54,55]. Cx43 can also enhance the transcriptional response to fibroblast growth factor 2 (FGF2) in osteoblasts by increasing PKC-δ activation [56], which is parallel with ERK signaling, ultimately regulating Runx2 activity. Beyond the synergetic impact on Runx, both ERK and PKC are required for the Cx43-dependent amplification of Runx2 activity induced by FGF2, but there is no intersectional phosphorylation between the two pathways. Activation of ERK is caused by intercellular communication signals, which mainly depend on the function of Cx43 and cell-to-cell contact, and the number of ERK-positive cells is increased when Cx43 is overexpressed [57]. Based on these discoveries, the Cx43 CT is suggested to be essential for the activation of ERK and has been shown to connect with PKC-δ via a specific domain [58,59]. The protein kinase A (PKA)-dependent pathway in osteoblasts is subordinate to the effect of Cx43, which is induced by sequestering β-arrestin after parathyroid hormone (PTH) stimulation [60] (Fig. 2).

Cx43 functions with specific molecules in osteoblasts. Specificity protein 1 (Sp1), one of the subordinate proteins downstream of the ERK pathway [55], is able to respond to dynamic Cx43 expression or activity and is recruited to osteoblast promoters, which ultimately leads to regulation of osteocalcin expression [60,61]. The function of specificity protein 3 (Sp3) is antagonistic to that of Sp1, and Sp3 can inhibit the transcription of osteocalcin [61]. The Cx43-dependent Sp1 pathway also promotes the transcriptional level of specificity protein 7 (Sp7)/osterix, which mainly acts to regulate osteoblastogenesis and enhance its recruitment to osteoblast promoters [60]. Similarly, Cx43 acts on Runx2 via the inositol polyphosphate/PKC-δ cascade [56,57,62] and was found to impact bone morphometric protein 2 (BMP2) and BMP2-triggered osteoblast mineralization in genetic ablation experiments [63].

#### 3.1.2 Cx43 in osteoclasts

Cx43-regulated GJs are also essential according to in vitro studies of osteoclast differentiation, preosteoclast fusion, osteoclastic bone resorption and osteoclast survival [64-66]. In vivo studies have demonstrated that general disruption of Cx43 channels is closely related to an uncontrolled status of the osteoclast surface, bone marrow and cathepsin K (osteoclast activity marker) [67]. Likewise, disruption of Cx43 at various stages leads to a rise in bone resorption and osteoclast number, resulting in a wide bone marrow cavity and external perimeter of long bones, as previously reported. Although supported by the given results in vivo, the direct role of Cx43 in osteoclastogenesis remains unclear. More importantly, these studies were conducted using whole-cell deletion of Cx43, which means that the Cx43-modulated GJs and HCs were generally influenced, which leads to difficulties in determining the true association of GJs or hemichannels with the experimental treatment consequences.

The effect of Cx43 on osteoclasts is not limited to cell-protein interactions. Results from in vivo experiments also revealed that the effect of Cx43 is related to different bone tissue environments, such as mechanical loading. Under mechanical loading, deletion of Cx43 increases osteoclastogenesis and bone resorption [41]. Conversely, under mechanical unloading, deletion of Cx43 attenuates the osteoclastogenesis induced by unloading and reduces the loss of trabecular bone, thereby inhibiting the effect of bone resorption [68,69].

#### 3.1.3 Cx43 in osteocytes

Osteocytes are important hubs in bone tissue remodeling and development [70], which involve bone formation and resorption conducted by osteoblasts and osteoclasts, respectively. The cell bodies of osteocytes are embedded in the bone lacunae and surrounded by a fluid-filled space, with long dendrites forming a network connecting neighboring osteocytes, osteoblasts and osteoclasts on the bone surface [71,72]. Osteocytes are derived from osteoblasts, and after the alteration, osteocytes still hold traits imparted by Cx43, among which the most prominent are the Cx43-HCs located on the cell body membrane of osteocytes and Cx43-GJs that participate in the connection between adjacent bone cells [70,73].

##### Cx43-Hemichannels

**Figure 3.**
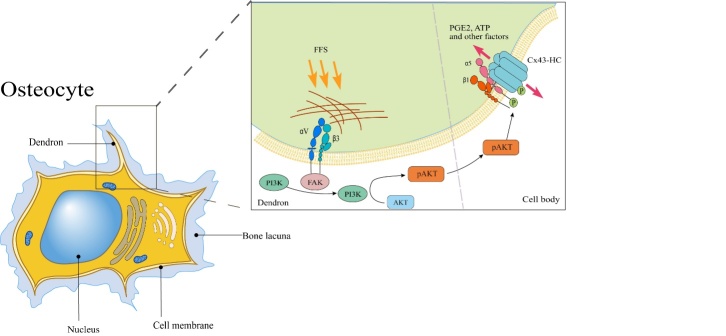
Mechanoreception associated with Cx43-HCs in osteocytes. FFS is transmitted through the ECM and glycocalyx at synapses, acts on integrin αVβ3 located in dendrites, and activates the PI3K/AKT signaling pathway through adherent FAK. Activated AKT directly phosphorylates Cx43 and the cytoplasmic domain of integrin α5 at the cell body membrane, causing integrin α5β1 to change conformation, ultimately leading to the opening of HC channels and allowing small bone anabolic factors such as pge2 and passage of ATP.

HCs are essential for the mechano-sensation capacity of osteocytes, especially Cx43-HCs. Cx43-HCs are dynamic channels that respond to various magnitudes and durations of flow shear stress (FSS) [73]. Thus far, the osteocyte mechano-sensation process related to Cx43-HCs has a relatively clear flow. On the basis of many studies, the mechano-sensation of bone cells is first assisted by deformation of the extracellular matrix (ECM) and transmission to the glycocalyx on the cell dendritic synapse, and then, the integrin αVβ3 located at the dendron receives an activation signal [74,75]. By binding intracellular proteins in the internal cytoskeleton to extracellular matrix proteins, integrin αVβ3 can be activated and stimulate PI3K/AKT signaling, mainly mediated by αV [6], which may draw support from FAK for signaling initiation [76]. Next, activated AKT directly phosphorylates both the cytoplasmic domain of Cx43 and integrin α5; although these two phosphorylation events can enhance the interaction between the two proteins, phosphorylation of Cx43 plays a predominant role in regulation [77,78]. A series of phosphorylation events causes the integrin to change its conformation, which eventually leads to opening of the HC and allows passage of small bone anabolic factors, such as PGE2 and ATP [73,79,80] (Fig. 3).

Together, the detailed process by which mechanical signals are transmitted involves many essential components, and if even one of these components does not function normally, then the mechanosensing capacity of osteocytes will be disturbed. However, integrin αVβ3 is not the only force receptor because integrin α5β1 can also function as a force receptor, which means that there is more than one delivery path, although αVβ3 is the main force receptor in the routine that opens Cx43-HCs [81,82]. In addition, closure of Cx43-HCs relies on phosphorylation of Cx43 conducted by the MAPK pathway, which is activated by PGE2 [80]. Although other channels may participate in the regulation, based on recent studies, Cx43 HCs are irreplaceable players because they are the ultimate effectors and impact the release of small bone anabolic factors [73].

##### Cx43-Gap Junction

GJs on osteocytes build sensitive communication with adjacent lacunae osteocytes and osteoblasts and transmit several effectors, such as calcium, ATP, PGE2 and cAMP [83,84]. By transducing biophysical signals from osteocytes to osteoblasts, GJs are important, and both the upregulation and downregulation of Cx43 are of great significance. Functional GJs are formed between osteocytes and osteoblasts in response to FFS by mediating the expression and phosphorylation of Cx43 [85,86], and were found to subsequently promote osteoblastic differentiation, while inhibition of GJ formation impaired the normal process [87]. In addition to its effect on bone tissue, blockade of Cx43 GJs results in decreased fast-twitch skeletal muscle contractile force and myogenesis [88]. With the exception of the abovementioned clear conclusions on Cx43 HCs and Cx43 GJs, most studies have not made a clear distinction between HCs and GJs, and a few experiments are related to both Cx43-HCs and Cx43-GJs in osteocytes (Table 1).

**Table 1:** Other animal studies on function of Cx43.

Scope of Cx43 Deletion	Species	Abnormal change	Conclusion	Ref.
Global	Cx43-/- mice	Delayed intramembranous and endochondral ossification, which are results of a generalized osteoblast dysfunction. In the skull, the combined osteoblast abnormality and the known migratory defect of neural crest cells result in craniofacial defects and nonclosure of the cranial foramen.	GJ communication mediated by Cx43 is functionally involved in skeletal development and in normal function of bone forming cells.	42
Global	R76W mice (dominant-negative mutant inhibiting only Cx43 GJ channels)	n/a	Great impairment of Cx43 HCs in osteocytes accelerates vertebral trabecular bone loss and increase in osteocyte apoptosis, and further suggest that Cx43 HCs in osteocytes protect trabecular bone against catabolic effects due to estrogen deficiency	43
Global	Cx43 Δ130-136 mice (dominant-negative mutant compromising both GJ channels and HCs)	1.Greatly decreased vertebral trabecular bone mass. 2.Significant increase in the number of apoptotic osteocyte and empty lacunae. 3. In its OVX model, osteoclast surfaces in trabecular and cortical bones were not increased.		
Osteocytes	Cx43fl/fl;DMP1-8kb-Cre mice	1.Had no impacts on bone anabolism induced with intermittent PTH administration. 2.Collagen maturation induced by intermittent PTH administration was impaired.	Osteocytic Cx43 expression is not necessary for the anabolic response to the intermittent PTH administration in male mice; but the Cx43 is involved partially in the effect of PTH on the bone matrix environment.	44
Osteocytes and osteoblasts	Cx43ΔOb-Ot/- mice	1.Increased osteocyte apoptosis in cortical bone. 2. Elevated endocortical resorption and periosteal bone formation.	Cx43 is essential in a cell-autonomous fashion for osteocyte survival and for controlling the expression of osteocytic genes that affect osteoclast and osteoblast function.	45
Osteocytes	Cx43ΔOt mice	1.Induce changes in femoral geometry and increase osteocyte apoptosis. 2. Increased periosteal bone apposition and endocortical bone formation/		

Cx43 also has an impact on the survival of bone cells and can generally increase cell activities and lifespan. Mice lacking Cx43 in osteocytes and osteoblasts exhibited increased osteocyte apoptosis in cortical bone, and mice with Cx43 specifically removed from osteocytes showed changes in femoral geometry along with increased osteocyte apoptosis, leading to some areas lacking viable osteocytes and increased periosteal bone apposition and endocortical bone formation [45]. In addition, selective deletion of Cx43 in osteoblasts or osteocytes resulted in an increased RANKL/OPG ratio, which stimulates increased osteoclastogenesis and bone resorption [41,46] potentially conducted by osteocytes [89]. Cx43 has been shown to help bone cells fight against damage or cell death caused by oxidative stress [90].

In summary, Cx43 is an important regulator of signal transmission between osteocytes, responses of osteocytes to external stimuli, and the responses of osteocytes to other cells. Thus, the absence of Cx43 leads to musculoskeletal disorders, and Cx43 is a potential core target for future treatment of chronic musculoskeletal diseases. For instance, combining the existing foundations described above may lead to enlightening effects on the treatment or understanding of the development of diseases, such as fractures, osteoarthritis and osteoporosis [91,92].

### 3.2 Cx43 in synovial membrane

The cavity of diarthrodial joints is enveloped by a fibrous sheath called the joint capsule, which contains joint fluid. The wall of joint capsules is composed of two fairly distinct layers: the external layer, consisting of a thick, dense connective tissue called the fibrous layer, and the inner layer, which is the synovial membrane or synovium. The synovial membrane contains in its superficial layer a unique cellular lining that is one to three cells deep and is called the synovial intima, which is generally known to consist of macrophage-like type A cells (type A cells) and fibroblast-like type B cells (type B cells). Type A cells are responsible for phagocytosing synovial fluid, while type B cells mainly produce hyaluronic acid and mucin to nourish and mechanically protect, respectively, the underlying articular cartilage.

GJs mainly exist between type A cells to transmit intracellular signals, and fewer are present between type A and type B and between type B cells [93]. Cx43-HCs are important in synovial cells and mainly mediate exchange of messengers, such as ATP and PGE2, to respond to any stimuli reaching the cell. Although the entire function of Cx43 and the final results of regulation of Cx43 in synovial cells remain unclear, some studies have shown interesting connections that may uncover the role of Cx43 in the synovium. Matsuki and colleagues found that Cx43 and TNF-α mRNAs were strongly expressed in RA patient synovial tissue but expressed at low levels in OA synovia [7], indicating that the expression of Cx43 may be related to inflammation caused by TNF-α. However, in another study, the expression of Cx43 and the size and number of GJ plaques were found to be increased in OA patients [94]. In addition, ex vivo analysis of synovial biopsies from OA patients showed that IL-1β-stimulated generation of collagenase activity was reduced after pharmacologic inhibition of Cx43 [94,95].

It can be concluded that Cx43 in the synovium of OA patients is related to inflammation, and furthermore, findings have suggested that knockdown of Cx43 can reduce the transcription of some OA-associated catabolic genes [95-97], but the clear tendency in pathogenesis, regardless of OA or RA, is unknown.

### 3.3 Cx43 in cartilage

Cartilage consists of cartilage tissue and the surrounding perichondrium. Cartilage tissue is composed of chondrocytes and cartilage matrix, which is the extracellular matrix of cartilage tissue and determines the structural and functional characteristics of cartilage. Chondrocytes, which fill cartilage lacunae, are responsible for the development and maintenance of cartilage, the synthesis and secretion of matrices and fibers of cartilage tissue and generate a unique ultrastructure in terms of biochemical composition and biophysical properties during differentiation. The stratified ultrastructure of articular cartilage is usually divided into the superficial zone, middle zone and deep zone after differentiation [98]. Cx43 is expressed primarily in the superficial zone and gradually decreases from the middle to deep zones of the growth plate in humans [17]. Based on a report from Donahue [11], Cx43 mainly acts as a component of HCs in chondrocytes located in the superficial zone [17] and middle zone but primarily participates in the function of GJs in deep zones. The function of Cx43 in chondrocytes is versatile, including mechanotransduction, differentiation, proliferation and metabolic homeostasis [99-101].

According to differences in the fibers contained in the cartilage matrix, cartilage can be divided into three types: hyaline cartilage, elastic cartilage and fibro cartilage. To date, no reports have been published certifying the existence of Cx43-controlled connexons in elastic cartilage. However, in hyaline cartilage and fibrocartilage, specific connexins, including Cx32, Cx43, Cx45, and Cx46, have been reported to mediate the communication between chondrocytes and to be associated with the normal development of joints and some common diseases, such as OA [17,68,102-104]. In addition, a critical role for Cx43 has been observed in the mineralized fibrocartilaginous transition of bone attachment points in mouse tendons [105].

The existence of Cx43-HCs in cartilage was discovered in early research, and Cx43-HCs were the earliest proposed channel structure in the musculoskeletal system that responds to mechanical loading. Bruehlmann et al. found that Cx43 is also expressed in cells without physically attached adjacent cells, which may hint at a special function of hemichannels in these observed Cx43 locations [103]. Another study verified the presence of Cx43 in human cartilage and proposed that Cx43 was restricted to the superficial region of cartilage, down to approximately 200 µm below the surface [17]. Furthermore, compared with the previous conclusion that Cx43 is indicative of intercellular gap junction formation [106], research has provided evidence that Cx43 forms HCs in cartilage and chondrocytes in isolated culture [17]. HCs may play an indispensable role in cartilage because of their special association with PGE2. PGE2 release has been suggested to mediate mechanotransduction between chondrocytes [107,108]. Mechanical loading enables the generation of signals that can be transmitted to chondrocytes to trigger important metabolic cascades [107,109,110]. Although PEG2 may rely on two pathways to cross the plasma membrane, with one being the prostaglandin transporter (PGT) [111,112] and the other Cx43 hemichannels, PGT mainly controls the import of PGE2 rather than its release [113,114]. Although whether Cx43 hemichannels in cartilage act as the most important negotiable form of PGE2 release is unknown, Cx43 has been demonstrated to respond to mechanical stimuli and control the increasing release of PGE2 in osteoblasts [115,116] and osteocytes [117,118]. Furthermore, evidence has shown that the expression of Cx43 in OA cartilage increases and overtly surpasses that in controls [119]. Interestingly, mechanical loading in bone tissue is widely recognized to play an important role in OA, and a recent report showed that mechanical loading can promote OA through the gremlin-1-NF-κB pathway (gremlin-1, a newly detected mechanical loading-inducible factor in chondrocytes, can activate NF-κB signaling) [120,121]. In recent years, studies on Cx43-HCs in cartilage have explored the specific functions of HCs, and more detailed mechanisms of action and the potential application of HCs in diseases are not yet known. For GJs in chondrocytes, studies have suggested that intercellular communication among human chondrocytes may occur via GJ channels [119]. Some experiments have shown that Cx43 is expressed in animal chondrocytes; more importantly, Cx43 can form normal GJ channels but is only detected in dense monolayer and micro mass cultures [17,33,106,122-126]. However, Cx43-GJs are not only located in clusters of chondrocytes as previously thought [11], which means that distant chondrocytes in mature articular cartilage can connect with one another via special physical connections, and it was finally demonstrated that cell-to-cell communication between highly specialized and distant chondrocytes in cartilage occurs through GJ channels that are predominantly composed of Cx43, which allows for the exchange of various molecules, including ions, second messengers, miRNAs and other compounds, such as glucose and amino acids [126-128]. Interestingly, mature articular chondrocytes can differentiate into a special cell type that has gracile cytoplasmic arms extending from the cell body, and the arms contain Cx43-GJs to facilitate communication with distant chondrocytes; furthermore, experimental results revealed that individual chondrocytes possess at least two arms extending along the matrix and reach different lacunae [127]. Moreover, the response to Cx43-GJ channel stimuli declines with age [90,129], which may lead to disorder of the equilibrium that maintains skeletal and joint integrity. Based on previously studied GJ functions, Gago-Fuentes et al. successfully demonstrated that cells located in the bone, cartilage and synovium have the ability to communicate directly with other GJ channels formed by Cx43, especially between chondrocytes and osteocytes [130].

Furthermore, the CT of Cx43 in cartilage has been shown to be involved in many chondrocyte functions. It is widely known that the CT of Cx43 acts as a hub in the cellular signaling network, and this function is mainly due to specific domains on the CT. In cartilage, these domains have already been demonstrated to be associated with controlling the phenotype and normal behaviors of chondrocytes according to results obtained from four experimental groups: controls, heterozygous for deletion of Cx43, heterozygous for CT-truncated Cx43 and CT null mice. CT-truncated Cx43 chondrocytes showed an increased proliferation rate and decreased synthesis of collagen type II (Col2A) and proteoglycans, which are the two most representative components of the cartilage ECM, and synthesis of these components was also diminished in other mutant groups to varying degrees. These changes impacted the number of chondrocytes and diminished the surface area of the ECM [131], which may lead to changes in the function of GJs and potential alterations in mechanical properties.

Chondrocytes normally exist as secreted functional substances that maintain the normal structure and capability of cartilage, with low proliferation probability and high metabolic activity. However, Cx43 mutation changes their behavior, and the main factor is the CT of Cx43, which shows the importance of the CT and indicates that an unknown function of Cx43 itself may influence some disease processes by causing abnormal activities in chondrocytes.

There are many upstream pathways involved in the regulation of Cx43 expression, but only a few have been confirmed in chondrocytes. Connective tissue growth factor (CTGF) has been found to facilitate functional GJ intercellular communication in chondrocytes by activating the PI3K/Akt pathway, which enhances Akt phosphorylation and translocation, ultimately increasing Cx43 expression. Conversely, inhibiting the trigger function of CTGF also led to diminished Cx43 expression in chondrocytes [132].

### 3.4 Cx43 in the bone vasculature system

The vasculature system plays a critical role in bone development and in maintenance of bone homeostasis under both physiological and pathological conditions [133] due to its function in nutrient and oxygen transport. In bone, blood vessels are distributed throughout the entire tissue except in cartilaginous areas, such as growth plates [134-136], forming a network consisting of arteries and veins that are connected through capillaries. Bone marrow vascular niches are significant in supporting hematopoietic stem cells (HSCs) and mesenchymal stem cells (MSCs). Bone vasculature and bone marrow vascular niches are critical in orchestrating osteogenesis and hematopoiesis by secreting angiocrine factors [137]. Bone blood vessels are commonly divided into two types, type H vessels and type L vessels, based on the high (type H) or low (type L) expression of CD31 and endomucin. Type H (CD31+ Emcnhi) vessels are crucial in bone development, bone formation, bone repair, and bone remodeling [138-140].

In the bone marrow, endothelial cells (ECs) are critical in regulating hematopoietic and mesenchymal stem cells. ECs interact with osteoblasts and hematopoietic cells, which is fundamental in maintaining bone homeostasis [141]. Cx43 has been studied in ECs in the endocrine system, which is closely related to bone metabolism. CD31+ Emcnhi vessels are also significant in the angiogenesis of endocrine tissues, and Cx43 was suggested to negatively regulate CD31+ Emcnhi ECs. Chen, et al. [142] generated EC-specific loss-of-function mice (Gja1iDEC) and found profoundly increased blood vessels in the pancreas, along with CD31+ Emcnhi islet capillaries, in the mutant mouse pancreas compared with control littermates, revealing the negative regulatory function of Cx43 in vascularization and EC proliferation. This phenomenon was also observed in the testis and thyroid gland, while no significant changes were found in the adrenal gland and ovaries. These results indicated that Cx43 is likely a regulator in the bone vasculature system and plays a role in maintaining bone homeostasis, but the further physiological function of Cx43 should be investigated.

Bone vasculature undergoes striking changes with aging [143]. Blood flow is obviously decreased upon aging, with a decline in Notch signaling, which is critical for the formation of type H vessels [139]. During aging, perivascular niches are also decreased, which leads to decreased hematopoietic stem cell and osteoprogenitor cell numbers and bone formation [144]. In addition to a decline in capillary and vessel numbers, aging is also associated with a decline in endocrine glands. The alteration of Cx43 in ECs of bone and endocrine tissues is indicated to be closely related to pathological activities during aging.

Vascularization is essential in bone repair and regeneration after bone damage due to fracture [145], infection [146], osteoporosis [147], osteoarthritis [148] or osteonecrosis [149] and in response to bone loss due to primary bone malignancies or bone metastasis[150, 151] because blood vessels are disrupted in these bone disorders. Cx43 alteration is also indicated to play a significant role in these musculoskeletal disorders.

## 4. Cx43 in musculoskeletal diseases

Cx43 is also a key factor in the progression of some musculoskeletal diseases, and studies on Cx43 may provide novel therapeutic targets for some other diseases.

### 4.1 Cx43 in rheumatoid arthritis

Rheumatoid arthritis (RA) is a chronic inflammatory autoimmune disease influenced by both genetic and environmental factors and is characterized by synovitis, which leads to destruction of articular cartilage and bone. Evidence derived from genetic, model and clinical studies indicates that immunopathogenesis is the main factor driving rheumatoid arthritis [152].

Cx43 has been demonstrated to be expressed in synovial tissues [93], and to have a crucial function in the regulation of immune inflammatory processes [153]. In experiments in a rat model of RA, after silencing of Cx43 expression, the tendency toward joint damage in diseased rats was suppressed, and the inflammation at the joint was reduced [96,154]. More convincingly, upregulation of Cx43 expression was found in the synovial tissue of patients with RA, and this upregulation was regulated by tumor necrosis factor-alpha (TNF-α) stimulation. When human rheumatoid arthritis synovial fibroblasts were transfected with Cx43 siRNA, the upregulation of TNF-α and IL-6 gene expression induced by TNF-α was significantly inhibited [7]. These studies combined with previous studies of physiological conditions demonstrate a role of Cx43 in promoting RA development and herald its potential as a therapeutic target.

### 4.2 Cx43 in osteoporosis

Osteoporosis is a disease characterized by pathologic changes in bone tissue, such as low bone mass and destruction of bone structure, which lead to decreased bone strength and an increased risk of fracture. Most studies on osteoporosis focus on hormones and age. In recent years, some studies have proposed a relationship between Cx43 and hormones, which suggests that Cx43 is possibly a key factor in osteoporosis treatment.

In Cx43-deficient mice, periosteal and endosteal expansion, cortical thinning, and increased porosity were prominently observed, which are associated with bone density and bone structure [41,155]. This indicates that changes in Cx43 are likely to cause osteoporosis-related symptoms. Parathyroid hormone (PTH) is commonly known to regulate bone metabolism through osteoclasts, and its main function is to promote bone resorption. However, one study showed that PTH can help osteoblasts resist apoptosis by affecting the accumulation of intracellular cAMP, which is based on Cx43-GJs, and ultimately promote bone mineralization regulated by osteoblasts [156]. Reduced Cx43 expression in osteoblasts may lead to incomplete mineralization of the extracellular matrix, and a certain degree of incomplete mineralization may be one of the causes of osteoporosis. More interestingly, the reduction in extracellular matrix stiffness further resulted in a decrease in the number of dendrites and the number of intercellular Cx43-GJs in single osteocytes and simultaneously affected the normal function of Cx43-HCs [91]. Changes in osteocyte dendrites and plasma membrane Cx43 affect cell-to-cell communication capabilities and the osteocyte-specific stress-sensing mechanisms mentioned above, further impairing the balance of osteocyte networks and ultimately leading to some of the hallmarks of osteoporosis. Correspondingly, Cx43 is also an important factor in the treatment process. In vivo experiments have demonstrated that bisphosphonates can prevent the apoptosis of osteoblasts and osteocytes during treatment, and this anti-apoptotic effect is critically dependent on the presence of sufficient levels of Cx43 in osteoblasts and osteocytes [157]. However, contradictory experimental conclusions have been reported, indicating that a lack of Cx43 is beneficial to the enhancement of the bone anabolic response to mechanical loading and can inhibit the bone decomposition response caused by mechanical unloading [158]. Combining these results, it can be inferred that the role of Cx43 in the development and treatment of osteoporosis is important and complex. Future work should try to identify the functions of the three main factors (Cx43, HCs and GJs), from pathological and therapeutic aspects and determine the relationship between Cx43 and age-related bone loss.

### 4.3 Cx43 in osteoarthritis

Osteoarthritis (OA) is the most common joint disorder, with hallmarks of age-related degeneration, joint swelling and limited mobility. OA is primarily characterized by failure of the repair process in damaged cartilage and is considered a disease of the entire joint, with all the surrounding tissues affected because of their physical and functional associations [159]. Chondrocytes and synovial fibroblasts in the affected area of OA show high expression of Cx43 [8,119,127,160]. Ben and his colleagues found that the CT of Cx43 binds tubulin in a direct manner via the juxta membrane (JM) segment, and this pattern may become a factor that is not negligible, affecting microtubules [13,161]. Subsequently, another study verified that the CT of Cx43 can indeed act on microtubules to affect the release of smad2/3, thereby regulating transmission of the downstream TGF-β signaling pathway [162]. Through this pathway, the high expression of Cx43 in osteoarthritic cartilage contributes to development of the disease [163] by disrupting the balance between Smad1/5/8 and Smad2/3 [162]. These findings indicate that limiting the accumulation of Cx43 on the cell membrane could be a new therapeutic approach to regulate healing after injury [164]. Compared with healthy human chondrocytes, the scaffold complex of Cx43 was remodeled in primary osteoarthritis chondrocytes, and the function of Cx43 was altered [165]. Cx43-GJs and Cx43-HCs in physiological quantities are involved in signaling mechanisms associated with intracellular calcium homeostasis, cell survival, cellular migration and remodeling. Under pathological conditions, the opening of excess Cx43-HCs is associated with propagation of inflammation and cell death-related signaling molecules. Altered Cx43-GJ-dependent responses may be associated with the inability to repair or regenerate tissue during chronic injury. Although it can be speculated that the normal function of Cx43 in cartilage and synovial cells may have a negative impact on the abnormal elevation of Cx43 in OA, there is still a lack of actual experimental results to support this hypothesis.

## 5. Conclusion

Numerous experiments have demonstrated the importance of Cx43 in the musculoskeletal system. During bone tissue development, growth, remodeling, and homeostasis maintenance, Cx43 plays a critical role as a communication hub, transmitting information between cells and helping cells sense and respond to stimuli from the extracellular environment. However, to date, there is still no clear conclusion on the detailed mechanism of Cx43.

**Figure 4.**
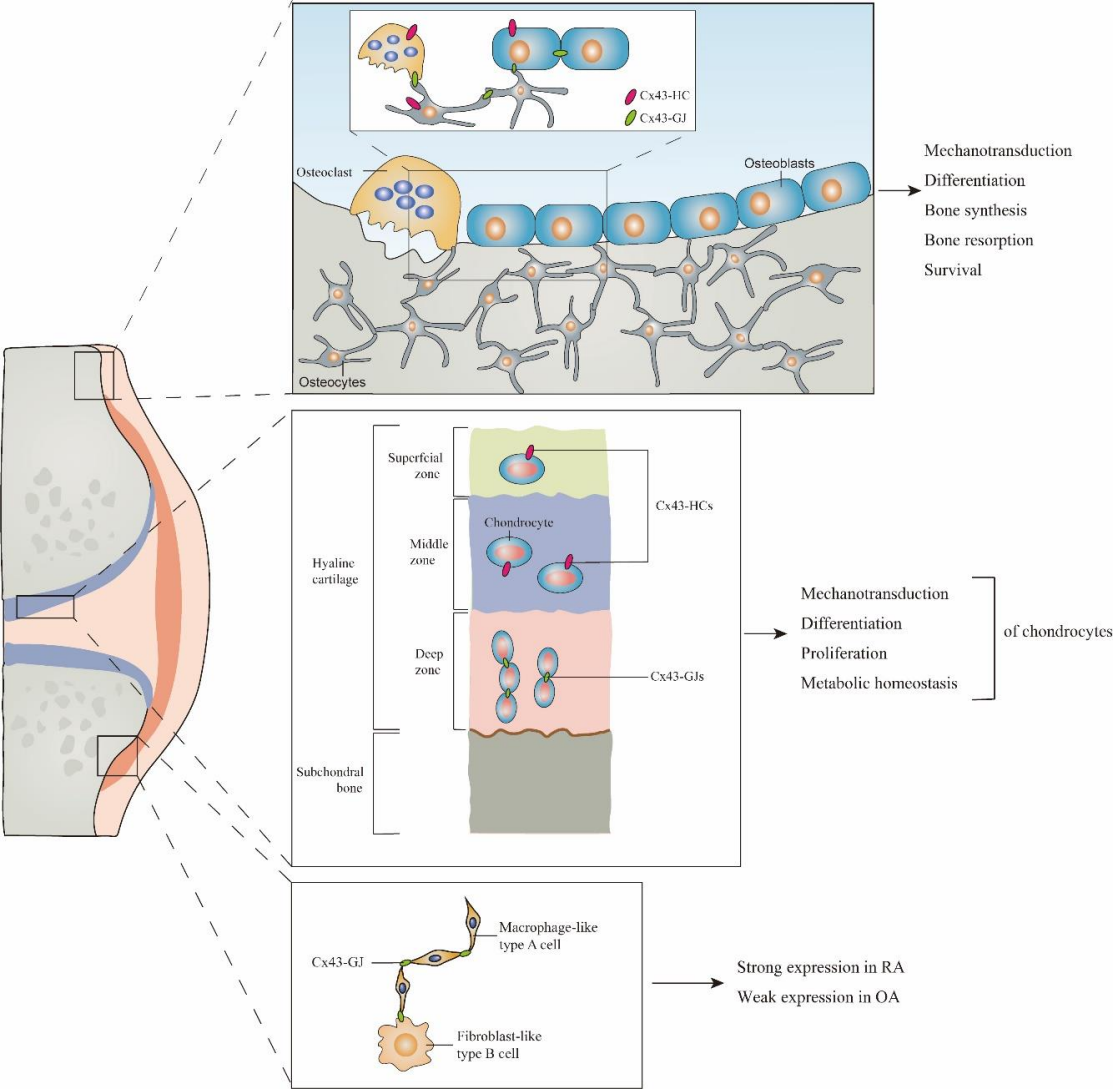
Cx43’s interactive roles between cells and tissues.

In recent years, breakthrough studies have revealed the role of Cx43 in hemichannels and the induction mechanism of osteocytes in response to extracellular fluid shear force. The induction mechanism of osteocytes in response to extracellular fluid shear force reveals a new concept, which not only involves the hemichannel model but also provides a new set of models that can replace the current models that have not led to identification of a new mechano-sensing direction for many years. Related research on Cx43-gap junctions is constantly being conducted and innovated based on the original theory. The regulatory mechanism underlying the permeability of gap junctions and the types of substances allowed to pass through are also constantly being unraveled. At the same time, gap junctions play an important role in the newly discovered mechanism of mechanosensing (Fig. 4).

However, there are still obvious bottlenecks in the current research. In most in vivo and in vitro experiments, a functional distinction between Cx43-HCs and Cx43-GJs has not been fully realized, and Cx43 knockout cells or mice exhibit complete deletion of Cx43-HCs and Cx43-GJs. Moreover, no detailed studies have revealed the connection between Cx43-HCs and Cx43-GJs.

Studies have started to explore the role of Cx43 in other motor system diseases, such as rickets, osteomalacia, and gouty arthritis. Some studies have shown that abnormal Cx43 expression is closely related to abnormal phenotypes in musculoskeletal disorders, but few studies have investigated and confirmed the detailed mechanism.

Cx43-directed therapy has not been proposed because the role of Cx43 in disease is unclear. However, according to the existing basic research, the role of Cx43 is dominated by chronicity, which controls a portion of the skeletal microenvironment and is a key in chronic disease progression. If the expression or function of Cx43 can be altered to adjust certain factors, such as the stress environment, osteogenesis and bone resorption balance, it may provide more effective therapy for related chronic diseases. Targeted therapies against Cx43 have appeared, such as α-connexin carboxyl-terminal peptide, which was developed to specifically target abnormal Cx43-HCs in cancer cells for the treatment of tumors [166]. In addition, antisense oligonucleotides against Cx43 and Cx43 blocking peptides have been used in clinical treatment [167,168]. According to these, Cx43 has the potential to become a targeted molecule in therapy and is an important object worthy of attention and research in the future.

In the future, the roles of HCs, mechanotransduction, and Cx43 in many other diseases will be further studied, leading to discovery of new priorities, including establishing efficient models, distinguishing components and the corresponding roles of linkers and HCs, and revealing more details regarding the physiological functions of Cx43.
